# From Haemorrhagic Shock to Years of Survival: A Case Report of PIK3CA-Mutated Estrogen Receptor-Positive and Human Epidermal Growth Factor Receptor 2-Negative (ER+/HER2-) Metastatic Breast Cancer With Satellite Lesions

**DOI:** 10.7759/cureus.91888

**Published:** 2025-09-09

**Authors:** Giorgi Vepkhishvili, Elene Zaalishvili, Luka Beridze, Zaza Aladashvili, Anano Nebieridze

**Affiliations:** 1 Oncology, Todua Clinic, Tbilisi, GEO; 2 Internal Medicine, David Tvildiani Medical University, Tbilisi, GEO; 3 Internal Medicine, Petre Shotadze Tbilisi Medical Academy, Tbilisi, GEO; 4 Medicine, Tbilisi State Medical University, Tbilisi, GEO

**Keywords:** alpelisib, case report, cutaneous metastases, hr positive/her2 negative, metastatic breast cancer, pik3ca mutation, ribociclib

## Abstract

Metastatic hormone-receptor-positive (HR+) human epidermal growth factor receptor-2-negative (HER2-) breast cancer remains the most common subtype of metastatic breast cancer, and is increasingly approached as a chronic condition. Genomic alterations in this disease subtype can create opportunities for targeted intervention, and advances in systemic therapy and radiotherapy have extended survival. However, real-world management continues to be shaped by treatment-related toxicities, cost considerations, and the evolving nature of tumour biology. We describe a 69-year-old Georgian woman who presented in haemorrhagic shock due to a neglected ulcerated breast mass and was subsequently found to have de novo stage IV HR+/HER2- metastatic breast cancer. She achieved a partial remission with induction docetaxel, but over three years she required multiple lines of systemic therapy, including sequential endocrine agents, cyclin-dependent kinase 4 and 6 (CDK4/6) inhibitor ribociclib, capecitabine, and eventually the PI3Kα inhibitor alpelisib following identification of an exon 9 PIK3CA mutation on next-generation sequencing. Alpelisib had to be discontinued because of severe hyperglycaemia. The case illustrates how repeated biopsies and genomic profiling can reveal actionable targets yet highlights the difficulties in balancing efficacy, toxicity, cost, and patient preferences. It also underscores the importance of multidisciplinary care, judicious use of palliative radiotherapy, and advocacy for equitable access to modern therapies.

## Introduction

Hormone-receptor-positive and human epidermal growth factor receptor-2-negative (HR+/HER2-) metastatic breast cancer (MBC) is defined by the expression of oestrogen and/or progesterone receptors without HER2 overexpression/amplification, which carries clear therapeutic implications. First-line endocrine therapy plus a cyclin-dependent kinase 4 and 6 (CDK4/6) inhibitor reduces the risk of progression or death by ~44% (hazard ratio ~0.56) versus endocrine therapy alone, establishing this approach as the standard of care [1]. Alterations in the phosphoinositide 3-kinase/protein kinase B/mammalian target of rapamycin (PI3K/AKT/mTOR) pathway, particularly phosphatidylinositol-4,5-bisphosphate 3-kinase catalytic subunit alpha (PIK3CA), enable targeted treatment. Newer agents, including inavolisib (selective PI3Kα) and capivasertib (AKT inhibitor), have shown additional benefit [2]. In PIK3CA-mutant disease, alpelisib prolongs progression-free survival (PFS) but carries notable metabolic toxicity [3]. These options are reflected in the updated 2025 recommendations [4]. Nevertheless, real-world uptake is constrained by economic and regulatory barriers [5]. We report a de novo HR+/HER2- case of MBC presenting in haemorrhagic shock with prolonged survival, illustrating how genomics, palliative radiotherapy, and access considerations shaped care.

## Case presentation

Initial presentation

**Figure 1:**
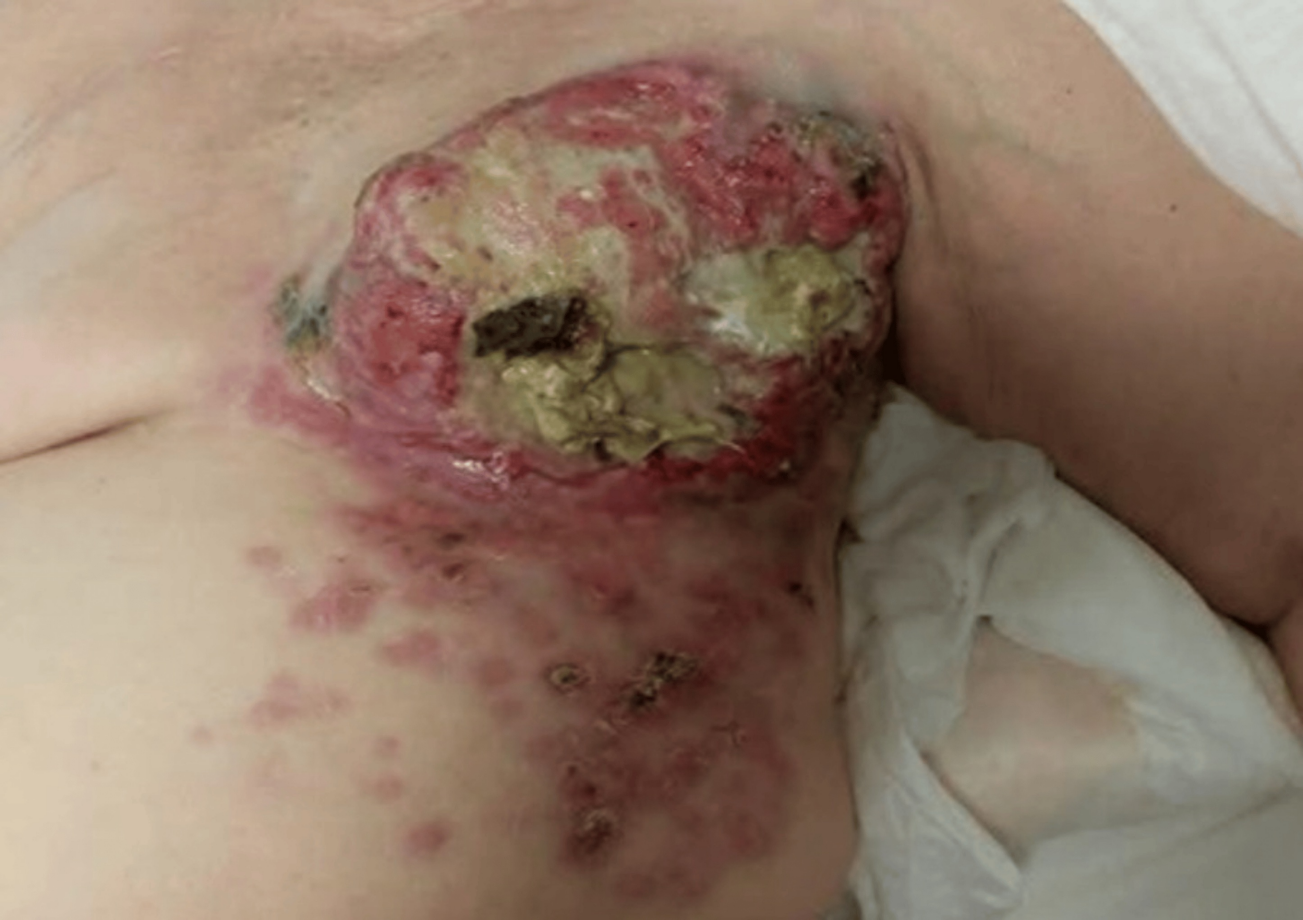
The image shows a large, ulcerated, bleeding breast mass with necrosis and friable tissue, surrounded by satellite nodules on inflamed skin The findings suggested an aggressive local invasion with cutaneous spread.

A 69‑year‑old post‑menopausal woman from Georgia presented to the emergency department in November 2021 with haemorrhagic shock. She had a neglected 16 cm necrotic, ulcerated left‑breast mass that was actively bleeding (Figure 1).

**Figure 2:**
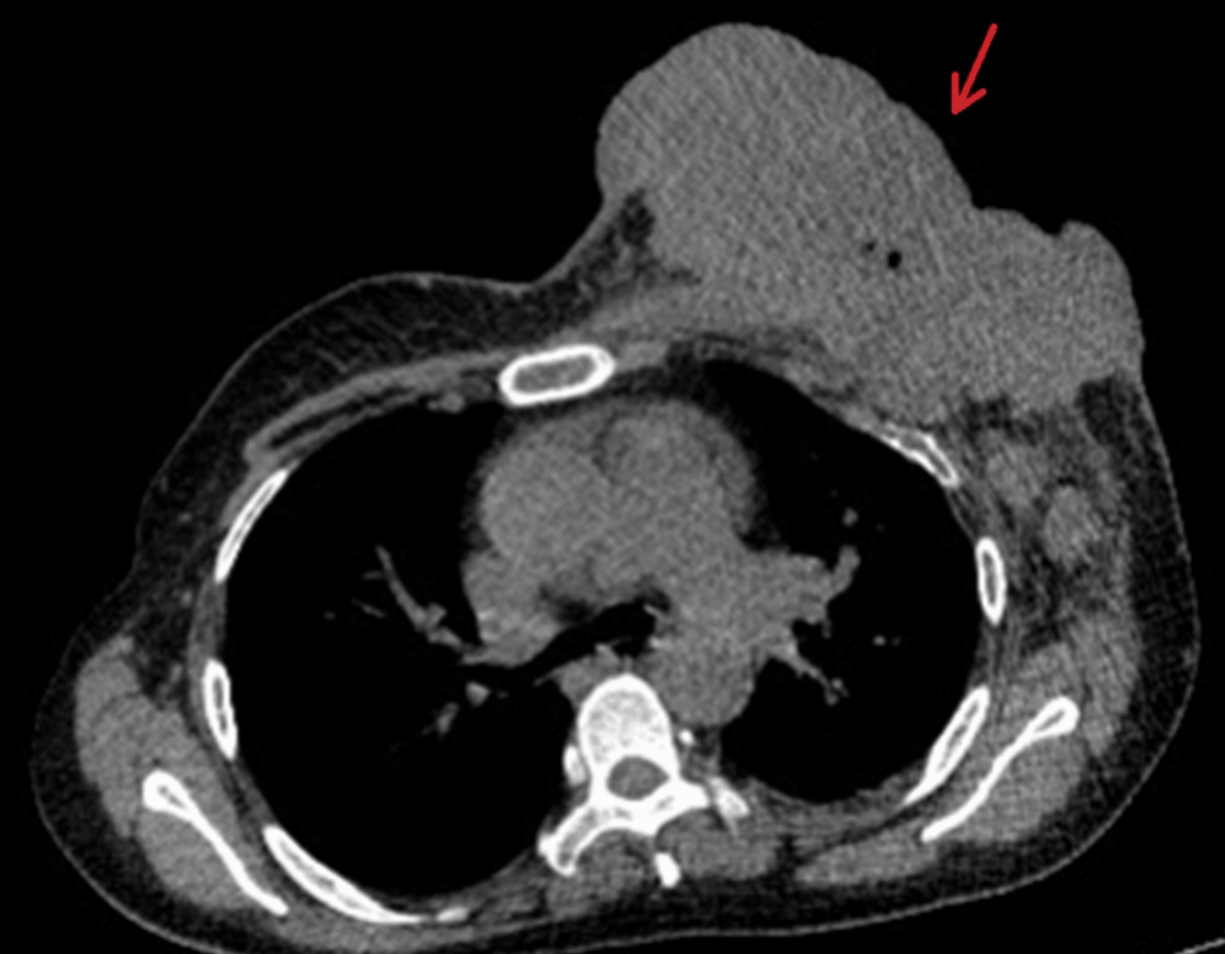
Axial contrast-enhanced CT of the chest showing advanced left breast malignancy A large, 16 cm multinodular mass was seen involving the left breast, with overlying skin ulceration and infiltration of the surrounding soft tissue. Multiple enlarged axillary lymph nodes were present (10-40 mm), consistent with locally advanced breast cancer.

Examination revealed multiple ipsilateral axillary nodes (10-40 mm) and a lytic lesion in the right 11th rib on bone scan; there was no visceral disease (Figure 2).

**Figure 3:**
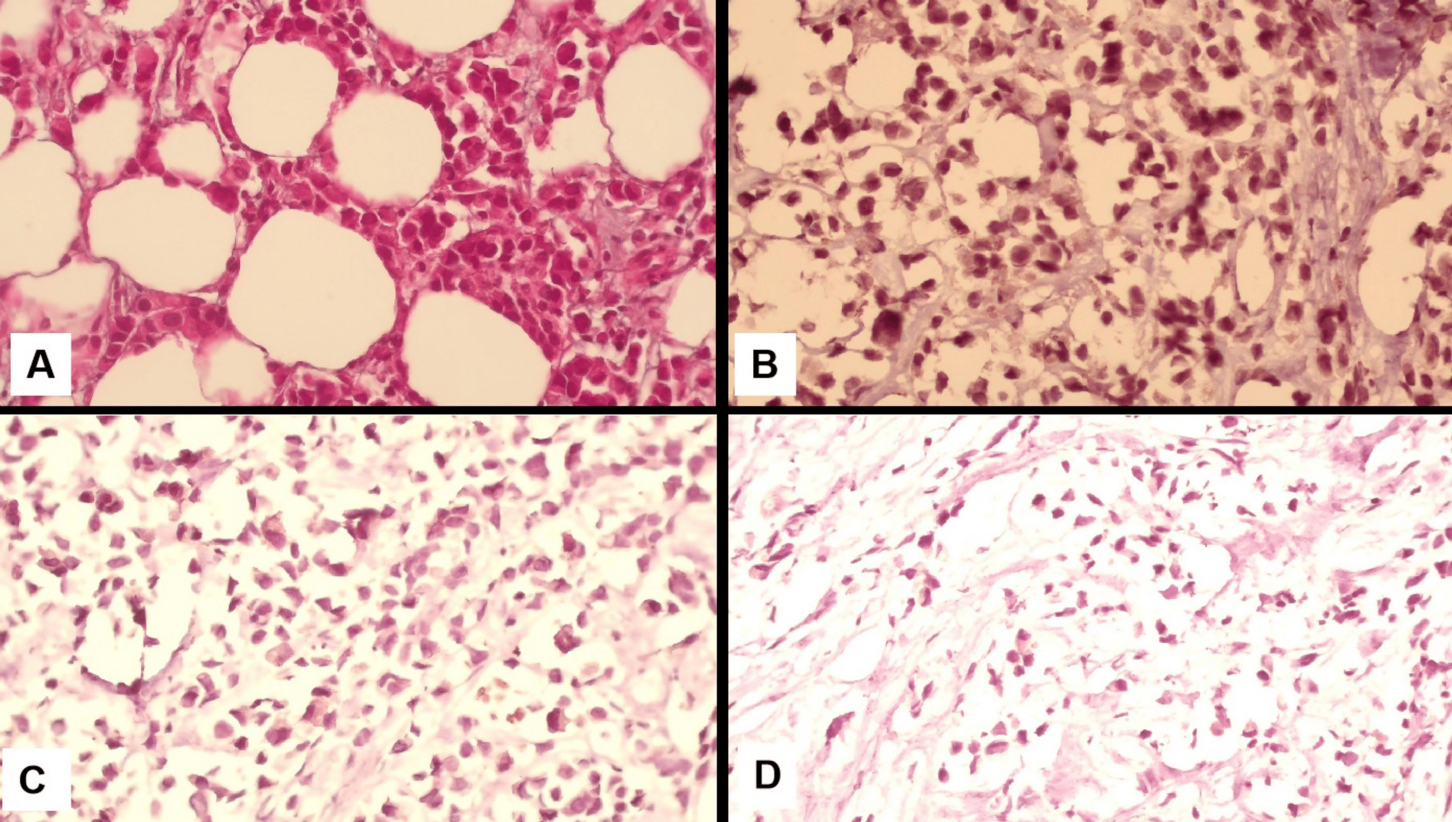
Biopsy results A. Hematoxylin and Eosin (H&E) staining of grade 3 invasive ductal carcinoma (400x magnification) showed infiltrating malignant epithelial cells with high nuclear pleomorphism, increased mitotic activity, and loss of glandular differentiation; B. Estrogen receptor (ER) immunohistochemical staining showed strong and diffuse nuclear positivity in the majority of tumour cells, indicating an estrogen receptor (ER)-positive status; C. Immunohistochemical staining for the progesterone Receptor (PR) at 400x magnification. The sample showed negative nuclear staining, consistent with PR-negative status; D. Immunohistochemical staining for human epidermal growth factor receptor-2 (HER2) showed absent membranous staining in tumour cells, consistent with a HER2-negative status.

Biopsy showed a grade 3 invasive ductal carcinoma that was oestrogen receptor (ER) positive (Allred score 6/8) [6], progesterone receptor (PR) negative, and HER2+ by immunohistochemistry with negative fluorescence in situ hybridization (Figure 3).

The Ki‑67 index was ~40%. Staging corresponded to T4bN2M1 (de novo stage IV) disease [7]. She received aggressive resuscitation with blood transfusions and underwent a core needle biopsy to confirm the diagnosis.

Induction chemotherapy

**Figure 4:**
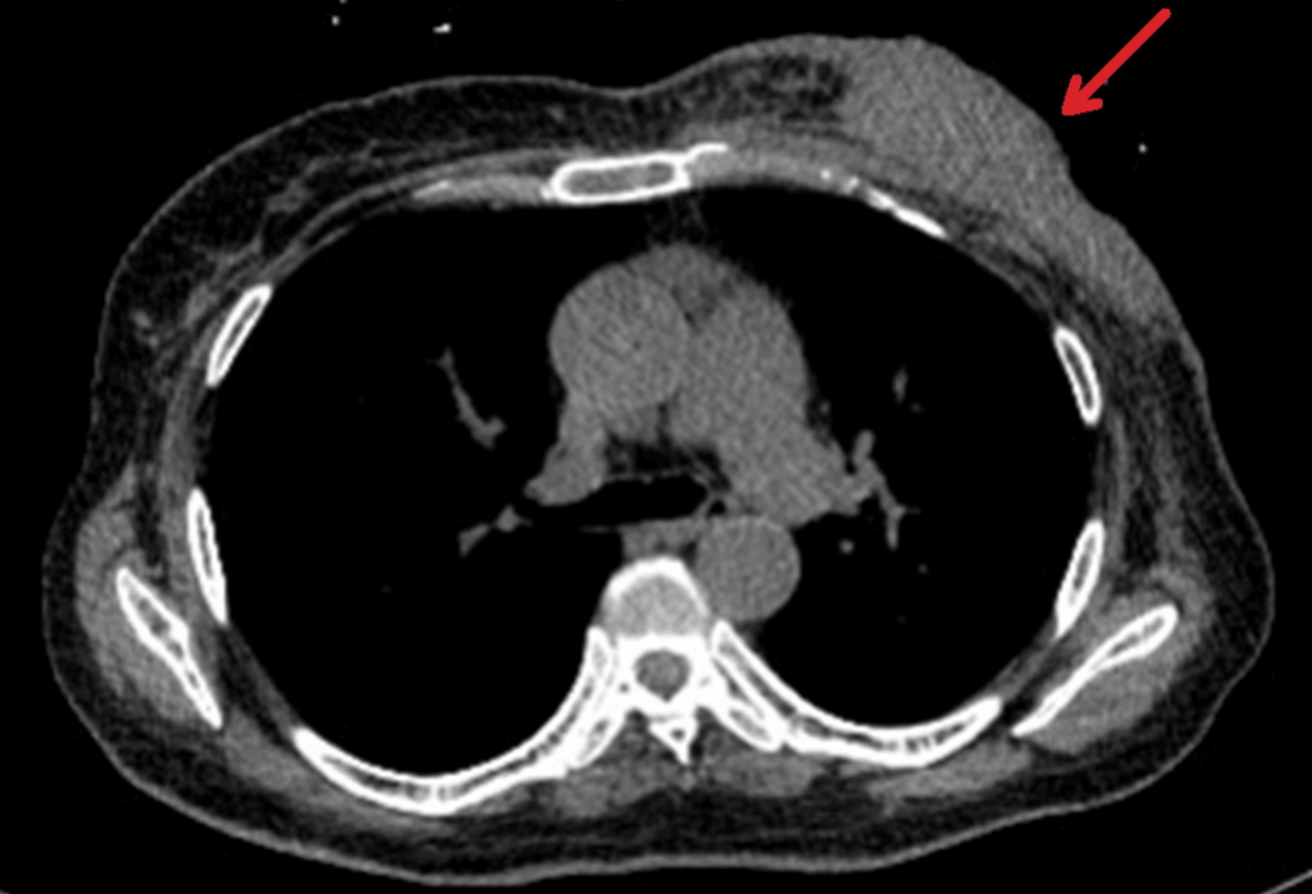
Axial CT demonstrating interval response to treatment The left breast mass had decreased in size to 67 mm, with reduction in axillary lymphadenopathy, and now measured 10-15 mm, consistent with partial treatment response. The arrow highlights the reduced tumor burden at the site of the satellite lesion compared with pre-treatment imaging, illustrating radiographic response.

Owing to the bleeding risk and high tumour burden, six cycles of docetaxel were administered from December 2021 to June 2022. The mass regressed markedly from ~16 cm to 6.7 cm and the axillary nodes decreased in size, while the rib lesion remained stable (Figure 4).

This chemo‑responsive component permitted the transition to endocrine therapy.

First‑line endocrine therapy 

In July 2022, a multidisciplinary tumour board recommended an aromatase inhibitor plus a CDK4/6 inhibitor. However, ribociclib was not reimbursed nationally in Georgia at that time, and the patient declined due to the cost. She was started on fulvestrant (500 mg intramuscularly every four weeks). Approximately one month later, she developed persistent bleeding and odour from the ulcerated tumour, so palliative radiotherapy (41.25 Gy in 15 fractions) with deep inspiration breath‑hold (DIBH) technique was delivered to minimise cardiac exposure. After radiotherapy, the bleeding and exudate diminished, and she resumed systemic therapy.

Subsequent disease progression

By June 2023, new cutaneous nodules arose outside the radiotherapy field. She was switched to letrozole (her first aromatase inhibitor) and received stereotactic radiotherapy to the new lesions. A repeat biopsy was recommended to reassess tumour biology, but she declined at that time.

Access to targeted therapy

**Figure 5:**
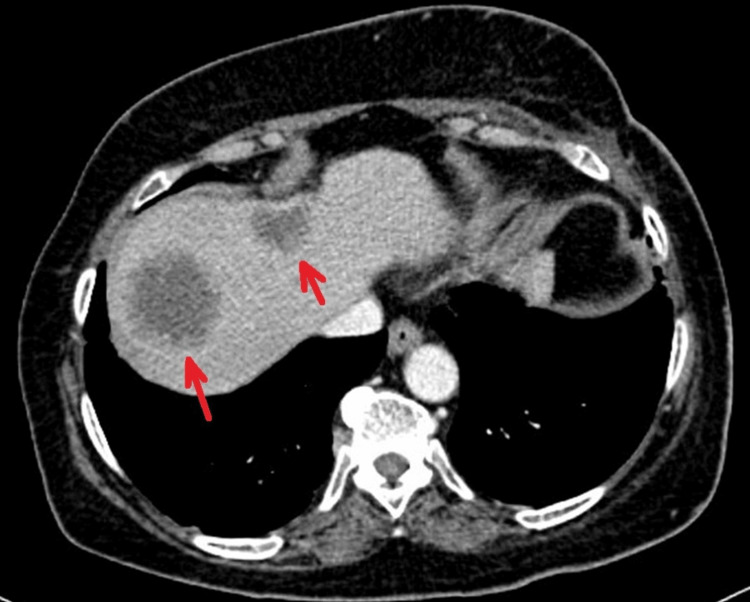
Axial non-contrast CT scan of the upper abdomen demonstrating two new hypodense lesions in the right hepatic lobe (red arrows), consistent with hepatic metastases

In August 2023, the national health system approved reimbursement for CDK4/6 inhibitors. She commenced anastrozole plus ribociclib (600 mg on a three‑week‑on/one‑week‑off schedule). The combination was well tolerated aside from mild neutropenia. In March 2024, restaging scans showed disease progression with new liver lesions (Figure 5), cutaneous metastases and abdominal wall nodules.

A liver biopsy was recommended, but she again declined and instead started capecitabine, achieving stable disease for several months.

Genomic profiling and targeted therapy

In October 2024, after radiographic progression, she consented to a core needle biopsy of the enlarging hepatic lesion. Histology confirmed metastatic breast carcinoma with low ER expression (~10% nuclei), negative PR and non‑amplified HER2. Next‑generation sequencing identified an exon 9 PIK3CA mutation (E542X). The PI3Kα inhibitor alpelisib was initiated in January 2025 together with fulvestrant. Unfortunately, within a month, she developed life‑threatening hyperglycaemic hyperosmolar syndrome. She required intensive care unit admission and insulin therapy, and alpelisib was permanently discontinued. The patient declined re‑challenge at a reduced dose or with metformin.

Current status

Since March 2025, she has been maintained on tamoxifen for residual ER expression, with slowly progressive disease in the liver and bones. She has undergone additional palliative radiotherapy for symptomatic lesions. As of spring 2025, more than three years from diagnosis, she remains alive with acceptable performance status, focusing on quality of life. Written informed consent was obtained for publication.

## Discussion

This case underscores several critical points in the management of metastatic HR+/HER2‑ breast cancer.

Role of genomics and precision therapy

Repeated biopsies and genomic profiling are essential because tumour biology can change during treatment. In the Epidemiological Strategy and Medical Economics (ESME) cohort, receptor status changed in a significant proportion of patients and re‑biopsy was recommended to confirm metastasis, assess prognosis, and identify new therapeutic targets [8]. Identifying a PIK3CA mutation in our patient enabled the use of alpelisib. The Clinical Studies of Alpelisib in Breast Cancer 1 (SOLAR‑1) trial demonstrated that alpelisib plus fulvestrant improved median PFS from 5.7 to 11.0 months versus fulvestrant alone [3]; however, grade 3-4 hyperglycaemia occurred in about 37% of patients, and 25% discontinued therapy due to adverse events [3]. Vigilant metabolic monitoring and prophylactic use of antidiabetic agents are crucial when initiating alpelisib in older or frail individuals.

Emerging targeted therapies

The patient’s inability to tolerate alpelisib highlights the need for alternative targeted strategies. Inavolisib is a highly selective PI3Kα inhibitor; the INAVO120 trial showed that inavolisib plus palbociclib and fulvestrant more than doubled PFS compared with palbociclib‑fulvestrant alone and led to an FDA approval in October 2024 [4]. The updated 2025 National Comprehensive Cancer Network guidelines recommend this triplet as a first‑line option for endocrine‑resistant PIK3CA-mutated MBC [4]. Capivasertib, an AKT inhibitor, also demonstrated significant improvement in PFS in the CAPItello‑291 trial (median PFS 7.2 vs 3.6 months; hazard ratio 0.60) and is now approved in combination with fulvestrant for patients with PIK3CA, AKT1 or Phosphatase and TENsin homolog deleted on chromosome 10 (PTEN) alterations [4]. mTOR inhibition with everolimus plus exemestane remains another validated option, prolonging PFS compared with exemestane alone [9]. Although these agents were unavailable to our patient in early 2025, awareness of such options is important for future therapy sequencing.

Importance of radiotherapy

Palliative radiotherapy remains invaluable for controlling symptomatic disease. Our patient initially presented with a bleeding, ulcerated tumour that responded well to a course of 41.25 Gy, consistent with reports that palliative chest‑wall radiotherapy reduces bleeding and foul odour and improves quality of life [10]. The DIBH technique should be employed for left‑sided irradiation whenever feasible because it decreases mean heart dose by 1-3 Gy and reduces high‑dose exposure [11]. Local radiotherapy is also useful for ablation of limited metastatic sites when systemic options are exhausted.

Health‑system and economic barriers

Even when effective therapies exist, access may be limited. Our patient initially refused ribociclib due to the prohibitive cost. In many low‑ and middle‑income countries, high‑cost medications such as CDK4/6 inhibitors remain inaccessible, leading to preventable deaths. A modelling study from Brazil estimated that the absence of ribociclib would result in 538 premature deaths among 4,294 premenopausal HR+/HER2‑ MBC patients over six years [5]. The same report emphasised that access to high‑cost drugs is a major challenge for cancer care and demands policy solutions [5]. The eventual approval of ribociclib reimbursement in Georgia allowed our patient to receive the recommended combination, highlighting how public policy can influence outcomes.

Multidisciplinary and individualised care

This patient’s prolonged survival reflects adaptive, patient‑centred management. Input from medical oncologists, radiation oncologists, intensivists, and other specialists guided therapy at each juncture. Treatments were adjusted based on tumour response, toxicity, access, and the patient’s preferences. Recognising patient autonomy, such as her decision to forgo re‑biopsy initially and later decline re‑challenge with alpelisib, was integral to maintaining trust and quality of life.

## Conclusions

This case illustrates the complexities of treating HR+/HER2‑ MBC in a resource‑constrained setting. Precision oncology and modern targeted therapies can offer meaningful extensions of survival, but their benefits must be balanced against toxicity, cost, and access. Genomic profiling enabled the use of a PI3K inhibitor, yet severe hyperglycaemia limited treatment. Advances like inavolisib and capivasertib promise better tolerance and efficacy and should be made accessible through national health programmes. Multidisciplinary management, timely palliative radiotherapy, and patient‑centred decision‑making were key to achieving more than three years of disease control and preserving quality of life in this case. Ongoing efforts to improve access to novel agents and supportive care will be essential as the therapeutic landscape for MBC continues to evolve.
